# Hydrogenated Germanene Nanosheets as an Antioxidative Defense Agent for Acute Kidney Injury Treatment

**DOI:** 10.1002/advs.202202933

**Published:** 2022-10-06

**Authors:** Zhixin Chen, Fenggang Qi, Wujie Qiu, Chenyao Wu, Ming Zong, Min Ge, Deliang Xu, Yanling You, Ya‐Xuan Zhu, Zhimin Zhang, Han Lin, Jianlin Shi

**Affiliations:** ^1^ State Key Laboratory of High Performance Ceramics and Superfine Microstructures Shanghai Institute of Ceramics Chinese Academy of Sciences Research Unit of Nanocatalytic Medicine in Specific Therapy for Serious Disease Chinese Academy of Medical Sciences (2021RU012) Shanghai 200050 P. R. China; ^2^ Center of Materials Science and Optoelectronics Engineering University of Chinese Academy of Sciences Beijing 100049 P. R. China; ^3^ Department of Clinical Laboratory Shanghai East Hospital Tongji University School of Medicine Shanghai 200120 P. R. China; ^4^ Shanghai Tenth People's Hospital Shanghai Frontiers Science Center of Nanocatalytic Medicine School of Medicine Tongji University Shanghai 200331 P. R. China

**Keywords:** acute kidney injury, covalent modification, germanene nanosheets, inorganic antioxidants, reactive oxygen species

## Abstract

Acute kidney injury (AKI) is a sudden kidney dysfunction caused by aberrant reactive oxygen species (ROS) metabolism that results in high clinical mortality. The rapid development of ROS scavengers provides new opportunities for AKI treatment. Herein, the use of hydrogen‐terminated germanene (H‐germanene) nanosheets is reported as an antioxidative defense nanoplatform against AKI in mice. The simulation results show that 2D H‐germanene can effectively scavenge ROS through free radical adsorption and subsequent redox reactions. In particular, the H‐germanene exhibits high accumulation in injured kidneys, thereby offering a favorable opportunity for treating renal diseases. In the glycerol‐induced murine AKI model, H‐germanene delivers robust antioxidative protection against ROS attack to maintain normal kidney function indicators without negative influence in vivo. This positive in vivo antioxidative defense in living animals demonstrates that the present H‐germanene nanoplatform is a powerful antioxidant against AKI and various anti‐inflammatory diseases.

## Graphical abstract

A novel paradigm is presented here for the biomedical applications of 2D H‐germanene‐based nanosheets, which exhibit intriguing antioxidative performance for broad‐spectrum reactive oxygen species (ROS) and excellent renal enrichment capacity. Combined with computational simulation, the underlying mechanism of 2D H‐germanene scavenging various ROS is investigated. The in vivo experiments show that 2D H‐germanene could effectively treat ROS‐induced acute kidney injury.

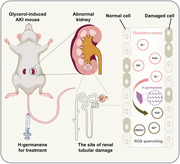

## 1 Introduction

Acute kidney injury (AKI), formerly known as acute renal failure, is mainly characterized by the deterioration of renal functions, including decreased glomerular filtration and abnormal accumulation of hematic nitrogenous waste.^[^ ^1^ ^]^ As a common complication of various diseases, such as tumors, uremia, and COVID‐19, AKI is associated with a high morbidity and fatality rate, especially for long‐term hospitalized and severely ill patients.^[^ ^2^ ^]^ At present, supportive treatments are commonly adopted, such as kidney dialysis and transplantation, according to the severity of the disease; however, highly effective protocols for AKI treatment and recovery are still unavailable.^[^ ^3^ ^]^

In an in‐depth study of AKI, researchers have found that it is accompanied by an abnormal increase in reactive oxygen species (ROS), which cause kidney damage, and is considered to be the most probable key cause of AKI.^[^ ^4^ ^]^ Therefore, ROS clearance provides a new potential solution for AKI treatment.^[^ ^5^ ^]^ Several classic antioxidants used for inflammatory diseases, including acute liver injury and acute kidney injury, such as *N*‐acetylcysteine (NAC), amifostine, and acetyl‐l‐carnitine,^[^ ^6^ ^]^ exhibit therapeutic effects; however, these may induce various side effects, such as fast metabolism, low utilization, and low efficacy.^[^ ^7^ ^]^ Thus, biosafe material systems with excellent antioxidative capacity for ROS scavenging are highly desirable for AKI treatment.

The rapid development of nanomedicine has enriched existing methods for ROS scavenging. Various functional nanomaterials (such as ceria^[^ ^8^ ^]^ and melanin^[^ ^9^ ^]^) have been developed for the treatment of ROS‐related diseases with satisfactory efficacy.^[^ ^10^ ^]^ However, in addition to scavenging ROS, the materials used for AKI treatment should demonstrate a targeting ability for intrakidney enrichment. According to previous reports, flake‐like DNA frameworks possess kidney‐targeting functions for drug delivery.^[^ ^11^ ^]^ The compact layered structure of the DNA framework could escape the immune monitoring and enzymatic hydrolysis that are commonly observed in DNA regions in loose DNA assembly structures.^[^ ^12^ ^]^ In addition, the negatively charged DNA framework surface can reduce protein adsorption and subsequent protein corona formation,^[^ ^13^ ^]^ which are favorable for kidney‐targeting DNA frameworks.

As a new type of 2D nanomaterial, the geometrical framework of germanene resembles that of DNA, which could contribute to its passive accumulation in the kidney. Furthermore, germanene exhibits strong potential in biomedical applications, including tumor photothermal therapy, owing to its physical properties, such as morphology, size, electron transmission, and optical properties.^[^ ^14^ ^]^ Parallel with these promising applications, a growing interest has focused on the toxicology of nanoparticles. Nevertheless, germanene‐based nanomaterials have received less attention in biological applications than other materials, such as silica and ceria, and their antioxidation behavior by ROS depletion has not been studied extensively.

Hydrogenation is an effective method for tuning the bandgap of selected nanocatalysts for therapeutic applications, such as TiH_1.924_ nanodots^[^ ^15^ ^]^ for tumor sonodynamic therapy and H‐silicene^[^ ^16^ ^]^ for tumor photodynamic therapy. Hydrogenation changes the bandgap of germanene, making it a semiconductor with a direct bandgap.^[^ ^17^ ^]^ Such hydrogen‐terminated germanene (H‐germanene) can be employed as an electron donor to function as an antioxidant.

**Scheme 1:**
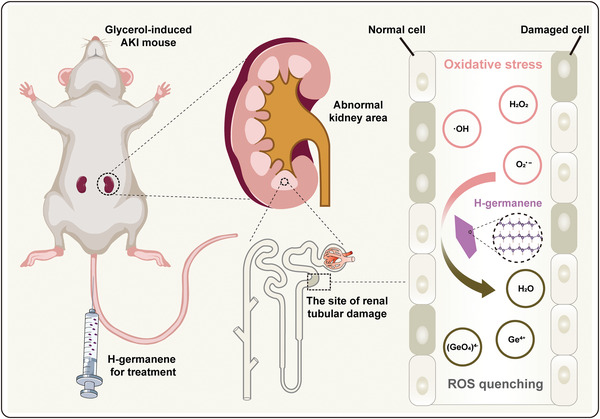
Schematic of H‐germanene as an ROS‐scavenging agent against AKI. H‐germanene injected through the caudal vein can be effectively enriched at the renal tubular damage site in the glycerol‐induced AKI mouse, further effectively scavenging ROS for AKI treatment.

Herein, we report the design and synthesis of 2D H‐germanene nanosheets (NSs) with a flake‐like DNA geometry, which can passively target the kidney and eliminate multiple ROS to treat related diseases, such as AKI (**Scheme** **1**). H‐germanene was found to have broad‐spectrum free radical scavenging, which can efficiently remove hydrogen peroxide (H_2_O_2_), superoxide anions (O_2_^•−^), and hydroxyl radicals (·OH), even at relatively low concentrations. In addition, the in vivo and in vitro experiments noted the positive effect of H‐germanene on AKI treatment without significant toxicity. As a new type of antioxidant, this inorganic nanomaterial features an NS‐like DNA framework for effective kidney accumulation, ROS scavenging, and resultant kidney protection against ROS damage for AKI treatment.

## 2 Result and Discussion

### 2.1 Synthesis and Characterization

**Figure 1:**
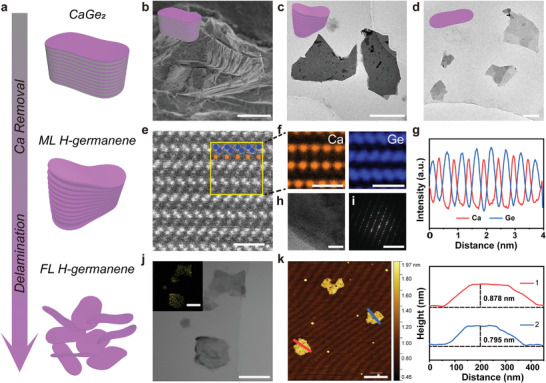
Synthesis and characterization of precursor CaGe_2_ and H‐germanene nanosheets. a) Schematic of the H‐germanene synthesis by etching of ${\left[Ca\right]}_{n}^{+}$ layers and subsequent ultrasound delamination. Multilayered germanene (ML H‐germanene) is formed after the removal of the Ca layer from the precursor CaGe_2_, which is exfoliated into single‐ or few‐layered germanene (FL H‐germanene). b) Scanning electron microscope (SEM) image of the precursor CaGe_2_ crystal. Scale bar: 15 µm. Transmission electron microscope (TEM) images of c) ML H‐germanene and d) FL H‐germanene. The thickness and sheet size are gradually decreased as required. Scale bar: c) 2 µm; d) 200 nm. e) High resolution (HR)‐TEM image, f) elemental mapping, g) corresponding elemental linear scanning profiles, and h) HRTEM image and i) SAED pattern of CaGe_2_. Scale bar: (e,f) 1 nm; (h) 5 nm; (i) 10 1/nm. j) TEM image (inset shows the elemental mapping) of the H‐germanene nanosheets. Scale bar: 250 nm. k) AFM image of the H‐germanene nanosheets and their corresponding height measurement. Scale bar: 400 nm.

H‐germanene covalently terminated with hydrogen atoms at the surface was synthesized through Ca layer deintercalation from the precursor Zintl‐phase CaGe_2_ crystals and subsequent delamination (**Figure** **1**a,b). The bilayer sandwich compound CaGe_2_ was synthesized by vacuum‐sealing pure Ca and Ge powders with fixed stoichiometric ratios in a quartz tube container, followed by annealing at 1000 °C for 18 h. The as‐prepared CaGe_2_ crystals were well crystallized according to the X‐ray diffraction (XRD) patterns (Figure S1, Supporting Information). Further low‐temperature (−40 °C) etching of the precursor with concentrated hydrochloric acid (HCl) could effectively extract the Ca atoms from the lattice to form a soluble by‐product of calcium chloride (CaCl_2_), in which the covalent Ca—Ge bonds were broken, and covalent Ge—H bonds were simultaneously established. The multilayered H‐germanene (ML H‐germanene) exhibited a typical planar morphology with a size of several micrometers (Figure 1c). To obtain freestanding, single‐, or few‐layered H‐germanene (FL H‐germanene), the as‐prepared ML H‐germanene was subjected to a probe ultrasonic treatment in an ice water bath. This decreased the lateral size to 100–400 nm and the actual thickness of the NSs to less than 1 nm, which is favorable for satisfactory in vivo intravenous circulation for potential clinical use (Figure 1d). This facile strategy for the fabrication of freestanding H‐germanene NSs achieved its desired morphology with promising scalable production, thereby fulfilling the strict demands in biomedical applications.

Based on the high‐resolution transmission electron microscopy (HRTEM) image (Figure 1e), the as‐fabricated bulk CaGe_2_ features a layered microstructure, alternating arrangement of ${\left[\mathrm{Ca}\right]}_{n}^{+}$ layers, and corrugated honeycomb ${\left[\mathrm{Ge}\right]}_{n}^{+}$ layers. The in‐plane microstructure of the germanene NSs is regularly corrugated, referred to as a buckled structure with a point group of the staggered form (*D3d*), which significantly differs from the planar hexagonal configuration of graphene. The corresponding element mapping shows the separation of the Ge layers due to the divalent cation of the calcium layers and warpage peculiarity of the 2D Ge plane (Figure 1f,g). Meanwhile, the HRTEM image and selected area electron diffraction (SAED) pattern show a well‐crystallized standard rhombohedral (R3m) structure of pristine CaGe_2_ (Figure 1h,i). The TEM image indicates that the CaGe_2_ block first underwent exfoliation and Ca removal into a large thick structure of ML H‐germanene by low‐temperature etching, and then further transformed into free‐standing and ultrathin FL H‐germanene via probe ultrasonic treatment. The TEM image and corresponding element mapping reveal a well‐preserved Ge NS morphology after Ca removal from the Zintl‐phase precursor (Figure 1j). A well‐defined 2D structure was created with a sheet thickness of <1 nm, as observed by atomic force microscopy (AFM) (Figure 1k). The infrared and Raman spectra confirmed the successful synthesis of H‐germanene (Figure S2, Supporting Information).

### 2.2 ROS Scavenging Mimicking and Mechanism

**Figure 2:**
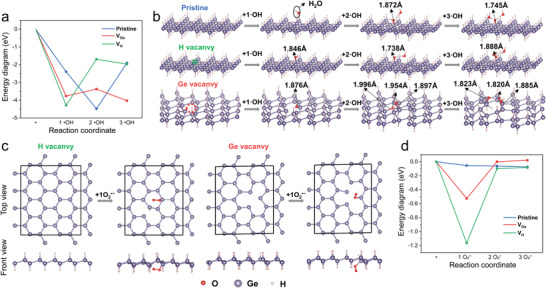
Theoretical calculation of ROS scavenging on H‐germanene. The DFT‐based molecular dynamic simulation of the interactions between different radicals (·OH, O_2_^•−^) and three nanosheet models including pristine/standard H‐germanene (noted as *p*H‐germanene), H vacancy‐containing H‐germanene (noted as H*‐germanene), and Ge vacancy‐containing H‐germanene (noted as H‐germanene*). a) Energy diagram and b) corresponding surface structure change of *p*H‐germanene, H*‐germanene, and H‐germanene* during ·OH scavenging. c) Geometrically optimized system during the O_2_^•−^ adsorption on *p*H‐germanene, H*‐germanene and H‐germanene*. d) Energy diagram of *p*H‐germanene, H*‐germanene, and H‐germanene* after O_2_^•−^oxidation.

H‐germanene‐enabled ROS decomposition is closely associated with the anti‐inflammatory efficacy in AKI alleviation. Therefore, simulating the ROS‐scavenging kinetics and thermodynamics of the monolayer germanene model is expected to be highly beneficial for understanding free‐radical chemistry. To understand the scavenging mechanism of the H‐germanene NSs interacting with ROS, density functional theory (DFT)‐based molecular dynamics (MD) simulations were performed. We constructed a standard 2D lamellar H‐germanene model with atomic‐level thickness to simulate 2D monoelemental Ge NSs. In addition, considering the defect introduction during liquid‐phase exfoliation and ultrasonic processing, pristine germanene NS models with H vacancies (denoted as H*‐germanene) and Ge vacancies (denoted as H‐germanene*) were established by deleting H or Ge atoms from the surface of the pristine/standard H‐germanene (denoted *as p*H‐germanene) monolayer. First, we explored the mechanism of H‐germanene and its defect‐related derivatives for ·OH scavenging. **Figure** **2**a,b shows the energy diagram of the gradual adsorption and bond length change of the atomic surface structure on the models of ·OH interacting with *p*H‐germanene, H*‐germanene, and H‐germanene*. In the *p*H‐germanene model, an ·OH radical spontaneously binds with the surface H atom to form an O—H bond with a bond length of 1.846 Å. The newly formed H_2_O molecule then escaped from the surface, leaving an exposed inner Ge atom. Subsequently, the second ·OH radical is adsorbed on the exposed Ge atom, followed by the adjacent third ·OH radical approaching the second ·OH, forming another H_2_O molecule, leaving an O atom anchored at the exposed Ge atom and a resultant Ge—O bond on the *p*H‐germanene surface. In the H*‐germanene model, the adsorption energy drops from −2 to −4 eV owing to the defects in the H vacancies (*V*_H_). The first ·OH radical is adsorbed on the Ge atom to form Ge—OH, followed by the same process as the *p*H‐germanene adsorption model. In the Ge vacancies (*V*_Ge_) marked in the H‐germanene* model, the ·OH radical tends to be adsorbed on the Ge around the Ge vacancies. According to the above results, the H‐germanene NS thermodynamically favors the adsorption of ·OH radicals, and the defect sites generated during the actual synthesis process further accelerate the quenching of ·OH radicals.

To investigate the antioxidative behavior of H‐germanene, we chose a typical ROS, O_2_^•−,^ as the simulation candidate. Figure 2c,d shows the structural changes and energy diagrams of the calculated O_2_^•−^ adsorption simulations on the H‐germanene surface. In the *p*H‐germanene model, O_2_^•−^ can only be physically adsorbed on the surface, and no chemical bond is formed between Ge and O, which can be regarded as the standard surface without exposed Ge atoms and saturated O—O bond. In comparison, O_2_^•−^ is chemically adsorbed on the H*‐germanene and H‐germanene* surfaces, thereby forming Ge—O—O bonds. In this process, 2D H‐germanene is oxidized as a reducing agent. In addition to the above two types of ROS, we also studied the adsorption behavior of H_2_O_2_ molecules on the *p*H‐germanene, H*‐germanene, and H‐germanene* surfaces, which exhibited comparable adsorption characteristics to those of O_2_^•−^ (Figures S3–S5, Supporting Information). Furthermore, we performed MD simulations on the reaction process of the H‐vacancy‐containing H‐germanene with H_2_O_2_ and ·OH (Figures S6 and S7, Supporting Information), in which the NS exhibited deformation and structural collapse with the exposure of more defect sites, thereby further favoring efficient ROS scavenging. Based on the above calculation results, the as‐synthesized 2D H‐germanene has scavenging capabilities for various ROS and is expected to be effective in the treatment of ROS‐related inflammation.

### 2.3 Antioxidative Efficiency Investigation

**Figure 3:**
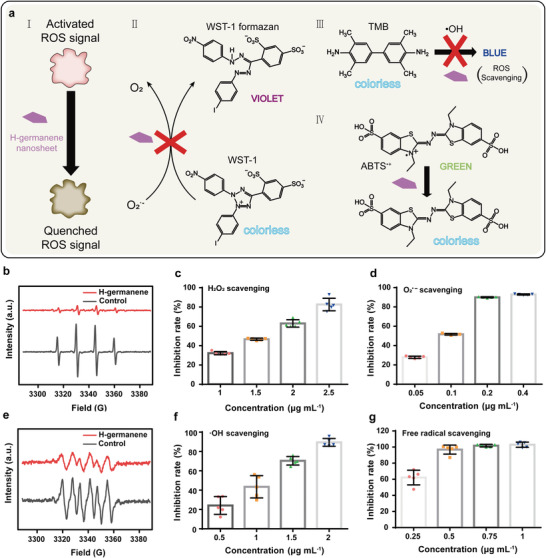
ROS‐scavenging activities of H‐germanene. a) Schematic of the chromogenic reactions, confirming the scavenging of ROS by H‐germanene. I) ROS elimination by H‐germanene. II) Transformation blockage over colorless WST‐1 to violet WST‐1 formazan by O_2_^•−^ depletion using H‐germanene. III) Chromogenic reaction prevention of 3,3′,5,5′‐tetramethylbenzidine through ·OH scavenging by H‐germanene. IV) De‐coloration of the green ABTS^+•^ solution in the presence of H‐germanene. b,e) ESR spectra of ·OH and O_2_^•−^ after reactions with H‐germanene. H‐germanene in scavenging in the presence of c) H_2_O_2_, d) O_2_^•−^, f) ·OH, and g) ABTS^+•^ (ppm = µg mL^−1^). Data presented are the means ± SD (*N* = 5).

Three representative ROS, namely H_2_O_2_, O_2_^•−^, and ·OH, were chosen to assess the ROS‐scavenging antioxidative activity of 2D H‐germanene NSs (**Figure** **3**a). A specific 1:2:2:1 multiple peak profile in the electron spin resonance (ESR) spectra demonstrated the presence of ·OH with a sharp decrease in its peak with the addition of H‐germanene, which indicates the high sensitivity of H‐germanene toward ·OH scavenging (Figure 3b). The peak intensity of O_2_^•−^ produced by the riboflavin photodegradation declined in the ESR spectrum upon the addition of H‐germanene, which indicates the effective consumption of O_2_^•−^ (Figure 3e). In addition, the dose‐dependent ROS‐scavenging effects of H‐germanene were quantitatively measured. First, we evaluated the consumption rate of H‐germanene in the presence of H_2_O_2_ (Figure 3c). An H_2_O_2_ inhibition rate of 80% for 2.5 µg mL^−1^ H‐germanene was obtained. The high reactivity of H‐germanene to H_2_O_2_ was also demonstrated by the change in the absorbance of the H‐germanene and H_2_O_2_ coexisting system over time (Figure S8, Supporting Information). Similarly, a high O_2_^•−^ inhibition rate of 90% by H‐germanene was achieved at a low concentration of 0.4 µg mL^−1^ (Figure 3d). The clearance rate of O_2_^•−^ exhibits a positive correlation with the H‐germanene concentration. Moreover, ·OH was almost completely depleted by H‐germanene at an equally low concentration of 2 µg mL^−1^ (Figure 3f). Compared with other nanomaterials used for ROS scavenging, such as CeO_2_ (60 µg mL^−1^),^[^ ^18^ ^]^ Au (100 µg mL^−1^),^[^ ^19^ ^]^ TiO_2_ (500 µg mL^−1^),^[^ ^20^ ^]^ and MnO_2_ (100 µg mL^−1^),^[^ ^21^ ^]^ H‐germanene with low concentrations functions as an emerging antioxidant.

To further confirm the antioxidative properties of H‐germanene, the total‐free‐radical‐scavenging performance was evaluated using the typical 2, 2′‐azinobis (3‐ethylbenzothiazoline 6‐sulfonate) (ABTS) radical cation decolorization assay. As shown in Figure 3g, H‐germanene enabled the reduction of ABTS^+•^ radicals, resulting in significant elimination of free radicals at a low H‐germanene concentration of 1 µg mL^−1^. Therefore, H‐germanene is expected to possess a strong ability to scavenge detrimental ROS. After H_2_O_2_ depletion by H‐germanene, the system gradually became transparent. However, after adding tannic acid, white precipitation (germanium tannate) occurred instantly (Figure S9, Supporting Information).^[^ ^22^ ^]^ Therefore, H‐germanene becomes soluble germanium ions, following the ROS reaction.

### 2.4 In Vitro Antioxidative Defense

**Figure 4:**
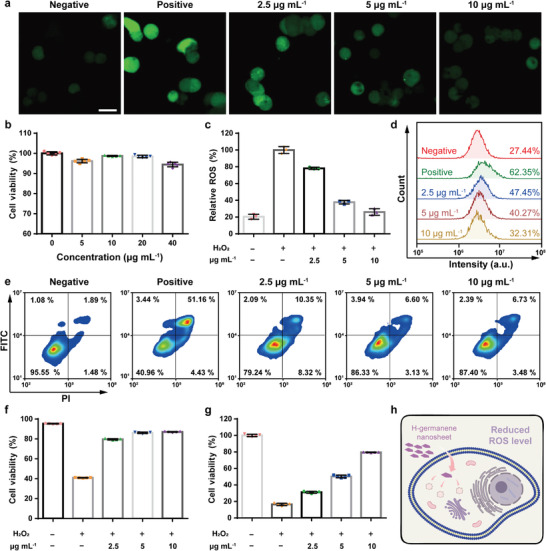
Cellular protective effect of H‐germanene against ROS damage. a) Confocal images of intracellular ROS signal (green fluorescence) and c) corresponding statistical analysis of the ROS level in the primary (Negative) and H_2_O_2_‐stimulated (Positive) HEK293 cells after different treatments for 24 h. Data presented are the means ± SD (*N* = 3). Scale bar: 100 µm. b) Viabilities of the HEK293 cells coincubated with different concentrations of H‐germanene for 24 h. Data presents the mean ± SD (*N* = 5). d) Statistical analysis of the ROS scavenging in H_2_O_2_‐stimulated HEK293 cells after different treatments for 24 h by flow cytometry. e) Flow cytometry analysis results of the apoptosis and necrosis of the primary (Negative) and H_2_O_2_‐stimulated (Positive) HEK293 cells after different treatments for 24 h and f) corresponding statistical analysis result. The data present the mean ± SD (*N* = 5). g) CCK‐8 kit tests of ROS scavenging in H_2_O_2_‐stimulated HEK293 cells after different treatments for 24 h. The data present the mean ± SD (*N* = 5). h) Schematic of the intracellular ROS scavenging based on H‐germanene. ****P < 0.0001; ***P < 0.001; n.s., no significance, one‐way analysis of variance (ANOVA).

Given the favorable antioxidative properties of H‐germanene, we evaluated its potential to scavenge ROS in vitro. As a vital component of renal metabolism, renal tubules are vulnerable to oxidative stress during AKI progression, which contributes to further renal dysfunction.^[^ ^23^ ^]^ Therefore, protecting the renal tubules against ROS damage is of great significance for alleviating AKI. The human embryonic kidney 293 (HEK293) cell line was used to verify the effects of H‐germanene against ROS‐induced cell damage in vitro. Here, intracellular oxidative stress was induced by H_2_O_2_ stimulation. Cellular ROS imaging and semiquantitative determination were performed using the ROS indicator 2′,7′‐dichlorofluorescein diacetate (DCFH‐DA), which converts into 2′, 7′‐dichlorofluorescein upon ROS‐induced deacetylation. As shown in the **Figure** **4**a,c and Figure S10 in the Supporting Information, the primary HEK293 cell line as the negative control features a low cellular ROS level that could be detected even with the weak signals by confocal laser scanning microscopy (CLSM). Upon H_2_O_2_ stimulation (250 µM), the cells show significantly increased signal ROS intensity, which was used as the positive group. The H‐germanene treatment groups present marked dose‐dependent ROS‐scavenging effects attributed to the favorable antioxidative activity of H‐germanene. According to the cell counting kit‐8 (CCK‐8) assay results, H‐germanene exhibited negligible cytotoxicity toward HEK293 cells, even at an elevated concentration of 40 µg mL^−1^ (Figure 4b). Flow cytometric analysis further revealed the dose‐dependent antioxidative defense of H‐germanene after co‐incubation with HEK293 cells for 24 h (Figure 4d). Furthermore, the quantitative flow cytometric results showed the H‐germanene NSs demonstrated an attractive antioxidative activity for preventing ROS‐induced apoptosis and necrosis even at an elevated H_2_O_2_ concentration of up to 500 µM (Figure 4e,f). Owing to the H‐germanene treatment, the cellular apoptosis and necrosis induced by H_2_O_2_ are significantly suppressed, confirming the marked cytoprotective effect of H‐germanene against ROS damage. Subsequently, we evaluated the cytoprotective effect of H‐germanene on cells using a CCK‐8 kit (Figure 4g). The H_2_O_2_ stimulation induced a series of damaging events, such as increased intracellular ROS level and subsequent cascade of pathological processes. However, the H‐germanene pretreatment can effectively regulate the ROS level, which confirms the strong potential of H‐germanene for AKI treatment (Figure 4h).

### 2.5 In Vivo Therapeutic Efficacy on AKI

**Figure 5:**
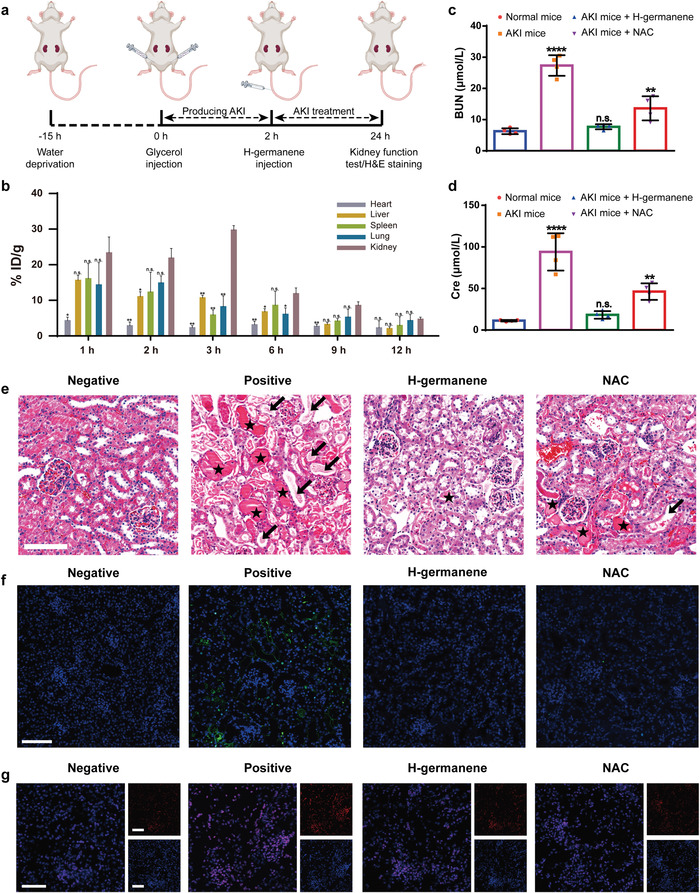
Therapeutic efficacies of H‐germanene on AKI mice. a) Scheme of the model establishment and treatment of AKI mice. The mouse AKI model was established by the intramuscular injection of glycerol in both hindlimbs after pretreatment by water deprivation for 15 h. In 2 h after injection, DSPE‐PEG‐modified H‐germanene was administrated for AKI treatment. b) Remaining Ge concentration in the main organs (heart, liver, spleen, lung, and kidney) of the mice at different time points after the tail vein injection of H‐germanene, which was quantitatively detected by ICP‐OES. The data present the mean ± SD (*N* = 3). Serum levels of the indicators in AKI mice 24 h after receiving different treatments: c) BUN and d) Cre. The data present the mean ± SD (*N* = 4). e) H&E staining of the kidney tissues from different groups. The arrows point to the damaged renal tubules and the asterisks indicate the formative casts. Scale bar: 100 µm. f) Terminal deoxynucleotidyl transferase deoxyuridine triphosphate nick‐end labeling staining of the kidney tissues from different groups. Green fluorescence represents cellular apoptosis. Scale bar: 100 µm. g) Fluorescence images of dihydroethidium (DHE) and 4',6‐diamidino‐2‐phenylindole (DAPI) costained kidney tissues from different groups. Blue: nucleus. Red: superoxide. Scale bar: 100 µm. ****P < 0.0001; **P < 0.01; *P < 0.05; n.s., no significance, one‐way ANOVA.

Owing to the desirable antioxidative defense behavior of H‐germanene in cellular evaluation, we further investigated the antioxidative capacity of H‐germanene nanomedicine in the AKI murine model. A mouse AKI model was established by rhabdomyolysis through water deprivation and intramuscular injection of 50% glycerol in both hind limbs. Various substances produced during the model establishment, such as myoglobin, can be enriched in the kidney through internal circulation and metabolism, resulting in renal oxidative damage and abnormal renal function (**Figure** **5**a).^[^ ^24^ ^]^ First, the in vivo distribution of H‐germanene was studied. The inductively coupled plasma‐optical emission spectrometry (ICP‐OES) test results showed that H‐germanene was rapidly enriched in the kidneys of the mice with a high concentration maintained in the first 3 h, thereby enhancing the therapeutic effect on the damaged kidneys (Figure 5b). At the same time, the main organs of the mice after intravenous injection of Cy5 fluorophore‐labeled H‐germanene for 12 h were collected. H‐germanene maintained effective enrichment in the kidneys (Figure S11, Supporting Information), which can be attributed to the compact structure of the DNA‐like framework and negative surface charge owing to 1,2‐distearoyl‐sn‐glycero‐3‐phosphoethanolamine‐poly(ethylene glycol) (DSPE‐PEG) modification (Figure S12, Supporting Information). This can prevent H‐germanene from undergoing enzymatic digestion and circumvent the formation of protein corona due to protein adsorption in the blood, thereby realizing effective accumulation in the kidney, instead of the liver and spleen.^[^ ^25^ ^]^

Twenty specific‐pathogen‐free (SPF)‐level mice (4–6 weeks old) were divided into four groups (*N* = 5): untreated normal mice (Negative group), AKI mice receiving intravenous phosphate‐buffered saline (PBS) injection (Positive group), equal volume of PBS containing H‐germanene (H‐germanene group), or NAC (NAC group). First, the levels of two core indicators for renal function assessment in AKI mice, namely blood urea nitrogen (BUN) and serum creatinine (Cre), were recorded 24 h after the model preparation.^[^ ^26^ ^]^ The results showed that the BUN and Cre expressions in the AKI mice was significantly higher than that in healthy mice, indicating severe kidney dysfunction in the mouse model (Figure 5c,d). As expected, the intravenous injection of H‐germanene led to a distinct and marked recovery of kidney excretory function, which was mostly restored to that of healthy mice, whereas NAC group only showed partial amelioration of the symptoms.

Subsequently, we measured the expression of several biomarkers in the kidneys of AKI mice pre‐ and post‐treatment to further evaluate the feasibility of H‐germanene against AKI. The superoxide dismutase (SOD) level in the kidneys was recorded to illustrate the molecular protective mechanism of H‐germanene in neutralizing kidney ROS. As shown in Figure S13 in the Supporting Information, the SOD level of the AKI mice significantly differed from that of the healthy mice, which was largely alleviated by the H‐germanene treatment, thereby proving the desirable efficacy of H‐germanene in ROS scavenging, SOD level maintenance, and renal cell defense. The expression levels of heme oxygenase‐1 (HO‐1) and kidney injury molecule‐1 (KIM‐1), which are representative biomarkers associated with kidney injury, were also analyzed in the renal tissues (Figure S14, Supporting Information).^[^ ^27^ ^]^ Consistent with the results of the BUN and Cre tests, H‐germanene treatment mostly restored HO‐1 and KIM‐1 in the kidneys of the AKI mice to similar values to those of healthy mice. Furthermore, H‐germanene functions as an excellent antioxidant for significantly reducing renal 8‐hydroxy‐2′‐deoxyguanosine (8‐OHdG) and thiobarbituric acid‐reactive substances (TBARS), which are biomarkers of DNA damage and lipid peroxidation, respectively (Figure S15, Supporting Information). In addition, hematoxylin and eosin (H&E) staining of the kidney tissues can more intuitively display the pathological changes in the kidneys following H‐germanene treatment (Figure 5e). Large numbers of damaged areas were noted in the kidneys of AKI mice, not only renal tubular damage but also tubular structure formation by denatured protein deposition. However, the renal damage in AKI mice was largely alleviated by the H‐germanene treatment. Furthermore, substantial tissue apoptosis has been reported in the kidney during AKI. Therefore, we performed terminal deoxynucleotidyl transferase deoxyuridine triphosphate (dUTP) nick‐end labeling (TUNEL) staining analysis on the kidney of the AKI mice, revealing the severe tissue apoptosis, which could be effectively downregulated by H‐germanene (Figure 5f). To further verify the critical role of ROS in the development of AKI, the kidneys of the AKI mice were stained with dihydroethidium (DHE) to determine the superoxide levels (Figure 5g). We also performed a quantitative statistical analysis of the superoxide levels in the kidney slices from different groups (Figure S16, Supporting Information). The results showed a marked reduction in the superoxide levels in the kidneys of the AKI mice treated with H‐germanene compared to those of the untreated AKI mice, indicating the high therapeutic potential of H‐germanene NSs against AKI.

### 2.6 In Vivo Biocompatibility Evaluation

**Figure 6:**
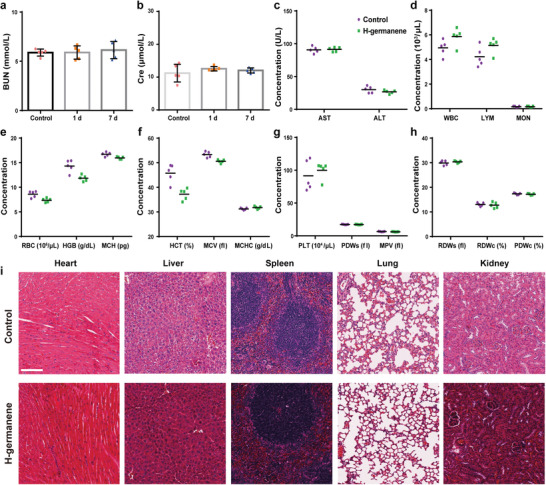
In vivo safety evaluation of H‐germanene. a) BUN and b) Cre concentrations after intravenous injection of H‐germanene and PBS on the first and seventh days. The data present the mean ± SD (*N* = 5). c–h) Hematological parameters of the mice 24 h after the intravenous injection of H‐germanene and PBS, respectively. The data present the mean ± SD (*N* = 5). i) In vivo toxicity of H‐germanene to the major organs (heart, liver, spleen, lung, and kidney) seven days after intravenous administration. Scale bar: 100 µm.

Subsequently, we injected high‐concentration H‐germanene into the tail veins of healthy mice to illustrate the in vivo safety of H‐germanene. After weight supervision for 7 d, the weight of all mice slightly fluctuated, proving the negligible adverse effect of H‐germanene on healthy mice (Figure S17, Supporting Information). The serum of the mice was collected at 1 and 7 d after the tail vein injection to measure the renal function indices of BUN and Cre, which exhibited minimal significant changes compared to the control group (**Figure** **6**a,b). In addition, we also tested the liver function and other hematological indices of the mice on 7 d after tail vein injection, which were within the normal range and consistent with the control (Figure 6c–h). Seven days after the intravenous injection of H‐germanene, the H&E staining of the main organs of the mice showed no significantly damaged areas (Figure 6i and Figure S18, Supporting Information). These results revealed the excellent biological safety and nontoxicity of H‐germanene, suggesting its potential for clinical applications.

## 3 Conclusion

In this study, we proposed and demonstrated a simple H‐germanene nanomedicine‐based AKI therapy strategy, featuring excellent kidney enrichment and renal protection. Computational simulation suggested the multiple ROS scavenging capability of H‐germanene, which was experimentally demonstrated to efficiently remove various ROS, even at relatively low concentrations. The in vitro experiments demonstrated that H‐germanene is highly biocompatible and cell‐protective against oxidative stress. The unique sheet‐like morphology of H‐germanene ensures its efficient kidney accumulation and facilitates its use as an antioxidative defense agent against AKI. Therefore, this simple formulated H‐germanene nanomedicine with broad‐spectrum ROS‐scavenging ability and unique kidney accumulation is highly promising as a new type of antioxidant for the treatment of AKI and various ROS‐related diseases.

## 4 Experimental Section

### Materials

Ca blocks (99.99%) and Ge powder (99.999%) were purchased from Acros. DSPE‐PEG was obtained from Shanghai Yare Biotech Co. Ltd. Sulfo‐cyanine5 succinimidyl ester was obtained from Shanghai Maokang Biotechnology Co. Ltd. CCK‐8, 3,3′,5,5′‐tetramethylbenzidine (TMB), and the total antioxidant capacity assay kit with a rapid ABTS method (T‐AOC Assay Kit) were purchased from Beyotime Institute of Biotechnology (Jiangsu, China). NAC, ammonium molybdate (AM), riboflavin, and FeSO_4_·7H_2_O were purchased from Sigma‐Aldrich Co. Ltd. (Shanghai, China). Aqueous HCl and H_2_O_2_ solutions were obtained from Sinopharm Chemical Reagent Co., Ltd. (Shanghai, China). The 5,5‐dimethyl‐1‐pyrroline *N*‐oxide (DMPO), SOD assay kit, reactive oxygen species assay kit, and Annexin V‐fluorescein isothiocyanate (FITC)/propidium iodide (PI) Apoptosis Detection Kit were purchased from Shanghai Dojindo Co., Ltd. Glycerol was obtained from Shanghai Yuncheng Co., Ltd. Enzyme‐linked immunosorbent assay (ELISA) kits (SOD Biochemical Detection Kit, TBARS Biochemical Detection Kit, HO‐1 ELISA Kit, KIM‐1 ELISA Kit, and 8‐OHdG ELISA Kit) were purchased from Shanghai YOBIBIO Co., Ltd. All the chemicals were used as received without further purification.

### Synthesis of H‐germanene NSs

a) CaGe_2_ Phase Synthesis. The compound was fabricated by the fusion of block calcium and powdered germanium pursuant to a molar ratio of 1:2 in a quartz tube. After loading, the quartz tube containing the raw materials endured the sequencing process, including evacuation to milliTorr pressures, subsequent sealing treatment with a hydrogen‐oxygen torch, final sintering process, annealing at 1000 °C for 18 h, and cooling down to ambient temperature for 2–3 d.

b) Synthesis of H‐germanene. The above products (CaGe_2_, 1 g) were immersed in 100 mL concentrated HCl aqueous solution (Sinopharm Chemical Reagents Co., Ltd., Shanghai, China) and stirred at a low temperature of −40 °C for 1 week. To improve the purity, the product was centrifuged, washed with water using ethanol, and finally dispersed in ethanol for further probe sonication. Sonication was performed in an ice‐water bath for 12 h using a sonic tip at 600 W for 3 s with an interval of 3 s.

### Surface Modification of H‐germanene with DSPE‐PEG

Freshly synthesized H‐germanene is unstable under physiological conditions. To guarantee the stability of H‐germanene when applied in vivo, 1 mg H‐germanene and 10 mg DSPE‐PEG were dissolved in 20 mL ethanol solution and reflux at 50 °C for 2 h. After centrifugation and washing three times with water, successfully modified H‐germanene‐DSPE‐PEG NSs with ideal dispersion were obtained. The H‐germanene used in the subsequent intracellular and in vivo experiments was modified using DSPE‐PEG. The mice with H‐germanene were labeled for fluorescence imaging. In the above modification process, 3% DSPE‐PEG was replaced with DSPE‐PEG‐Cy5. The subsequent steps were performed to obtain H‐germanene labeled with Cy5.

### Characterization of H‐germanene NSs

The morphology and composition of the CaGe_2_ and H‐germanene precursors were determined using a JEM‐2100F transmission electron microscope (200 kV), JEOL ARM‐300F with spherical aberration correction (300 kV), and a field‐emission Magellan 400 microscope (FEI Company). XRD was performed using a Rigaku D/MAX‐2200 PC XRD system (Cu *K*_*α*_, *λ* = 1.54 Å, 40 mA, and 40 kV). CLSM images were obtained using an FV1000 microscope (Olympus Company, Japan). AFM images were acquired using a Veeco DI Nanoscope Multi‐Mode V system. UV–vis–NIR absorption spectra were measured using a UV‐3600 Shimadzu UV–vis–NIR spectrometer. Quantitative analysis of the elements was performed using ICP‐OES (Agilent 725, Agilent Technologies, US). The ESR tests were performed using a JEOL‐FA200 ESR spectrophotometer. Infrared and Raman spectra were collected using a Nicolet iS10 FTIR spectrometer and LABRAM HR Evolution (532 nm), respectively.

### Calculation Method

The Vienna ab initio simulation package (VASP) was used to conduct spin‐polarized DFT calculations on the basis of the plane‐wave basis sets with the projector augmented‐wave method. The exchange‐correlation potential was addressed according to a generalized gradient approximation with the Perdew–Burke–Ernzerhof parametrization. Subsequently, the van der Waals correction of Grimme's DFT‐D3 model was conducted. A 4 × 2√3 × 1 supercell was constructed using hydrogenated germanene. A vacuum region of ≈20 Å was constructed to prevent the adjacent images from interacting. Brillouin‐zone integration was sampled with a Γ‐centered Monkhorst–Pack mesh of 2 × 2 × 1. The structures were completely relaxed until the maximum force on each atom less than 0.04 eV Å^−1^ and the energy convergent standard was 10^−6^ eV. The ab initio molecular dynamics (AIMD) simulations were conducted using a canonical ensemble (NVT) (parameters: 310 K, time step: 1.0 fs, simulation time: 15 ps). The adsorption energy of X (X = ·OH, O_2_^•−^, and H_2_O_2_) was calculated as *E*_b_(X) = E_*X_ − E_*_ − *E*_X_, where E_*_ is the energy of the hydrogenated germanene monolayer with an H atom removed, E_*X_ is the total energy of the monolayer with *X* adsorbed, and *E*_X_ is the energy of X.

### Measurement of Free Radical Scavenging

The ability of H‐germanene to scavenge free radicals was determined using the T‐AOC Assay Kit, through the color change as the developer. The assay was performed as follows. First, the ABTS and peroxidase working solutions were configured according to the manufacturer's instructions. Different concentrations of H‐germanene were fully mixed with the ABTS working solution in advance and then added to the peroxidase working solution. After incubation at room temperature for 6 min, the absorbance difference at 414 nm of the different systems was tested for calculating the scavenging efficiency of ABTS^+•^ following the equation: scavenging efficiency = [(*A*_1_ − *A*_3_) − (*A*_ge_ − *A*_2_)]/(*A*_1_ − *A*_3_) × 100%, where *A*_1_ is the absorbance of the mixture of ABTS and peroxidase working solutions, *A*_2_ is the absorbance of the H‐germanene dispersion, *A*_3_ is the absorbance of the buffer, and *A*_ge_ is the absorbance of the mixture of the H‐germanene dispersion, ABTS working solution, and peroxidase working solution. All tests were performed in quintuplicates (*n* = 5).

### Measurement of O_2_^•−^ Scavenging

a) SOD Assay Kit. An SOD assay kit was used to determine the ability of H‐germanene to scavenge O_2_^•−^. The H‐germanene dispersion was first added to the (2‐(4‐iodophenyl)‐3‐(4‐nitrophenyl)‐5‐(2,4‐disulphophenyl)‐2hydrotetrazole salt, disodium salt (WST) working solution, a highly water‐soluble salt that can react with O_2_^•−^, followed by the addition of xanthine oxidase, and finally full mixing (the final germanium concentrations of H‐germanene were 0.05, 0.1, 0.2, and 0.4 µg mL^−1^, respectively). After incubation at 37 °C for 20 min, the absorbances from different groups at 450 nm were determined, and the O_2_^•−^ scavenging efficiency of H‐germanene was calculated according to the following formula: scavenging efficiency = [(*A*_1_ − *A*_3_) − (*A*_ge_ − *A*_2_)]/(*A*_1_ − *A*_3_) × 100%, where *A*_1_ is the absorbance of the mixture of WST working solution and xanthine oxidase working solution, *A*_2_ is the absorbance of the H‐germanene dispersion, *A*_3_ is the absorbance of the WST working solution, and *A*_ge_ is the absorbance of the mixture of the H‐germanene dispersion, WST working solution, and xanthine oxidase working solution. All tests were performed in quintuplicates (n = 5).

b) ESR Test. O_2_^•−^ produced by the light irradiation of riboflavin (50 µL of 6 mM) was used to analyze the O_2_^•−^ scavenging ability of the H‐germanene dispersion (50 µL, 3 µg mL^−1^). DMPO functioned as a capture agent for O_2_^•−^ to form the adduct DMPO−O_2_^•−^, showing characteristic peaks in the ESR spectra. Changes in the characteristic peak intensities were used to evaluate the O_2_^•−^ scavenging ability of H‐germanene.

### Measurement of ·OH Scavenging

a) Microplate Reader. The scavenging activity of H‐germanene against ·OH was determined based on the effective removal of ·OH originating from the Fenton reaction. FeSO_4_ (1 mM) and H_2_O_2_ (2 mM) were mixed in sodium acetate buffer, followed by the addition of H‐germanene dispersion. After 5 min of reaction, TMB was used to measure the absorbance at 650 nm. All tests were performed in quintuplicates (*n* = 5).

(b) ESR Test. The ability of H‐germanene to scavenge ·OH produced by the Fenton reaction can also be demonstrated by the ESR spectra. The ·OH generated from FeSO_4_ (1 mM) and H_2_O_2_ (2 mM) was captured by DMPO, presenting peaks related to DMPO−OH adducts. Different concentrations of H‐germanene were added to the reaction system, and the ability of H‐germanene to scavenge ·OH was estimated according to the change in peak intensity.

### Measurement of H_2_O_2_ Scavenging

AM reacts with H_2_O_2_ to develop color for quantification. H_2_O_2_ was reacted with H‐germanene for 10 min, and then AM was added to test the absorbance of this system at 405 nm. All tests were performed in quintuplicates (*n* = 5).

### In Vitro Biocompatibility Evaluation of H‐germanene

The CCK‐8 assay was used to evaluate the cytotoxicity of H‐germanene. Briefly, HEK293 cells were first seeded into 96‐well plates at a density of 10^4^ cells per well, cultured in Dulbecco's modified Eagle's medium (high glucose) containing 10% fetal bovine serum and 1% penicillin/streptomycin, and then incubated in an incubator (37 °C, 5% CO_2_) for 24 h to ensure cell adhesion. Then, the cell culture medium was poured out, and fresh culture medium containing various concentrations of H‐germanene was added. After incubation for 24 h, two washes with sterile PBS were followed by the addition of 200 µL fresh culture medium and 20 µL CCK‐8 solution for a further 2 h incubation at 37 °C. Finally, cell viability was quantified by measuring the absorbance at 450 nm using a microplate reader.

### In Vitro ROS Scavenging Using H‐germanene

To demonstrate the ability of H‐germanene to scavenge ROS, HEK293 cells were seeded into 96‐ and 6‐well plates at a density of 1 × 10^4^ and 10 × 10^4^ cells per well, respectively. After 24 h of incubation, the medium was removed and fresh culture medium containing different concentrations of H‐germanene was added for incubation for an additional duration of 30 min. Subsequently, H_2_O_2_ at a concentration of 250 µM was added and incubated again at 37 °C for 24 h. To qualitatively and quantitatively describe the content of the intracellular ROS under H_2_O_2_ stimulation, DCFH‐DA was used to stain intracellular ROS. DCFH‐DA has no fluorescence and can pass freely through the cell membrane. After entering the cell, DCFH‐DA is hydrolyzed by intracellular esterases to generate DCFH. However, DCFH cannot penetrate the cell membrane, making it easy for the probes to be loaded into the cells. Intracellular ROS can oxidize nonfluorescent DCFH to generate fluorescent DCF. DCF fluorescence can be used to determine intracellular ROS levels. The cells incubated with H_2_O_2_ for 24 h were washed twice with serum‐free medium and then DCFH‐DA dye working solution was added for another 30 min. Finally, the unbound DCFH‐DA probe was removed by washing twice with a serum‐free medium. Because of the combined DCFH‐DA probe, the intracellular ROS could be imaged by laser confocal imaging and flow cytometry. Wells without H_2_O_2_ addition were considered as negative controls, whereas wells with H_2_O_2_ addition were considered as positive controls.

The H_2_O_2_ concentration was increased to 500 µM and the cytoprotective effect of H‐germanene was further illustrated by recording the cell viability, and ratio of apoptosis and necrosis. For the cells seeded in the 96‐well plates, after incubation for 24 h with 500 µM H_2_O_2_, the cells were washed twice with PBS, added with 200 µL fresh medium and 20 µL CCK‐8. After incubation at 37 °C for 2 h, the absorbance at 450 nm was tested to quantify the cell survival rate. For cells seeded in six‐well plates after incubation for 24 h with 500 µM H_2_O_2_, the cells were harvested and washed twice with cold PBS. Next, 5 µL Annexin V‐FITC and 5 µL PI were added to the cell suspension for another 15 min dark incubation at room temperature (25 °C). Finally, the binding buffer was added to the cell suspension at the end of the incubation period for the quantitative analysis by flow cytometry.

### Animal Treatment

Female institute of cancer research (ICR) mice were obtained from the Charles River Laboratories. All animal studies conformed to the guidelines of the Animal Care Ethics Commission of the Shanghai Tenth People's Hospital, Tongji University School of Medicine (Number: SHDSYY‐2020‐Z0026).

### In Vivo Biocompatibility Evaluation of H‐germanene

To illustrate the in vivo biocompatibility of H‐germanene, H‐germanene was injected into the tail vein of ICR mice (aged 4–6 weeks, 20–25 g) at a dose of 2 mg kg^−1^. The mice in the control group were intravenously injected with PBS. At 1 and 7 d after injection, the blood samples from the mice were collected for the serum biochemical tests and blood analysis. Serum biochemical tests were performed to detect two routine indicators of the renal function, namely Cre and BUN, and two routine indicators of the hepatic function, alanine aminotransferase (ALT) and aspartate aminotransferase (AST) levels. In addition, the major organs (heart, liver, spleen, lung, and kidney) of the mice sacrificed on 1 and 7 d were also collected and stained with H&E. The mice were weighed and recorded daily.

### AKI Mouse Models

The ICR mouse model of AKI induced by glycerol was constructed as previously reported.^[^ ^6^ ^]^ Briefly, the mice were first deprived of water but still had free access to food. After 15 h, the normal water supply was restored and 50% glycerol (6 mL kg^−1^) was injected intramuscularly into both hindlimbs.

### In Vivo Biodistribution of H‐germanene

To study the in vivo distribution of H‐germanene in the major organs (heart, liver, spleen, lung, and kidney), glycerol‐induced AKI mice were intravenously injected with 2 mg kg^−1^ H‐germanene. At various time points postinjection (1, 2, 3, 6, 9, and 12 h), the mice were sacrificed, and major organs were collected, weighed, and digested with aqua regia, followed by membrane filtration to quantify the H‐germanene enrichment of the different organs at different time points.

### In Vivo Therapeutic Outcome of H‐germanene in AKI Mice

To demonstrate the effectiveness of H‐germanene in AKI treatment, the mice were divided into four groups: normal mice, AKI mice, AKI mice with intravenous injection of H‐germanene, and AKI mice with intravenous injection of NAC. At 24 h after injection, the mice were sacrificed, and the blood samples were collected to quantify the levels of the renal function indicators BUN and Cre by serum biochemical tests. The left kidneys of all collected mice were homogenized for the detection of kidney biomarkers using various ELISA kits. An SOD assay kit was used to detect the SOD renal levels. The expression levels of KIM‐1 and HO‐1, which are two vital kidney injury biomarkers, were detected using the KIM‐1 and HO‐1 ELISA kits, respectively. In addition, 8‐OHdG and TBARS ELISA kits were used to determine the DNA damage and lipid peroxidation.

The right kidneys of the harvested mice were equally divided into two halves: one was used for H&E staining after the first fixation with 4% paraformaldehyde and subsequent embedding in paraffin; and the other was made into an optimum‐cutting‐temperature (OCT) specimen, according to previously reported strategies.^[^ ^6^ ^]^ OCT specimens were stained with DAPI and DHE for confocal microscopy to determine ROS in the kidney region.

### Statistical Analysis

All the statistical data were presented as the mean ± standard deviation (SD) from at least three independent experiments (*N* = 3). Cell viability was normalized to the control group. The significant differences in the experimental data were evaluated using one‐way ANOVA with Tukey's significant difference post hoc test (using OriginPro 8.5 software). The calculated probability (*p*) was distinguished as (*P < 0.05), (**P < 0.01), (***P < 0.001), and (****P < 0.0001).

## Conflict of Interest

The authors declare no conflict of interest.

## Supporting information

Supporting Information

## Data Availability

The data that support the findings of this study are available from the corresponding author upon reasonable request.
