# Supplementary material for: Hydrogenated Germanene Nanosheets as an Antioxidative Defense Agent for Acute Kidney Injury Treatment
Source: Adv Sci (Weinh). 2022 Oct 6;9(33):2202933. doi: 10.1002/advs.202202933 (PMC9685437; doi:10.1002/advs.202202933)
Supplement: Supplementary file 1 — Supporting Information [file ADVS-9-2202933-s001.pdf]

Supporting Information

Hydrogenated Germanene Nanosheets as an Antioxidative Defense Agent for Acute Kidney Injury Treatment

Zhixin Chen, Fenggang Qi, Wujie Qiu, Chenyao Wu, Ming Zong, Min Ge, Deliang Xu, Yanling You, Ya-Xuan Zhu, Zhimin Zhang, Han Lin,* and Jianlin Shi**

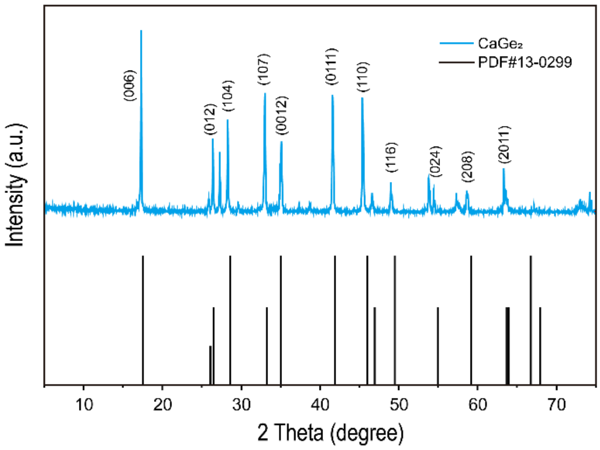

Figure S1. XRD pattern of precursor $CaGe_2$.

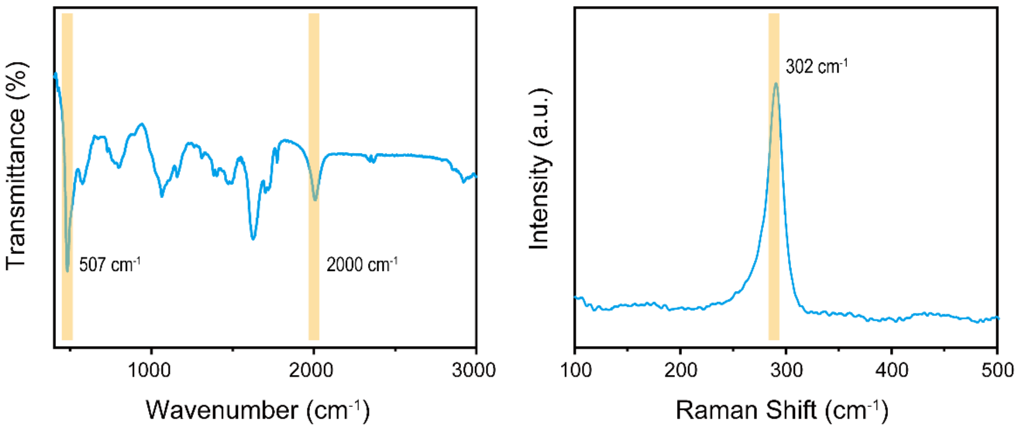

Figure S2. FITR and Raman spectra of H-germanene.

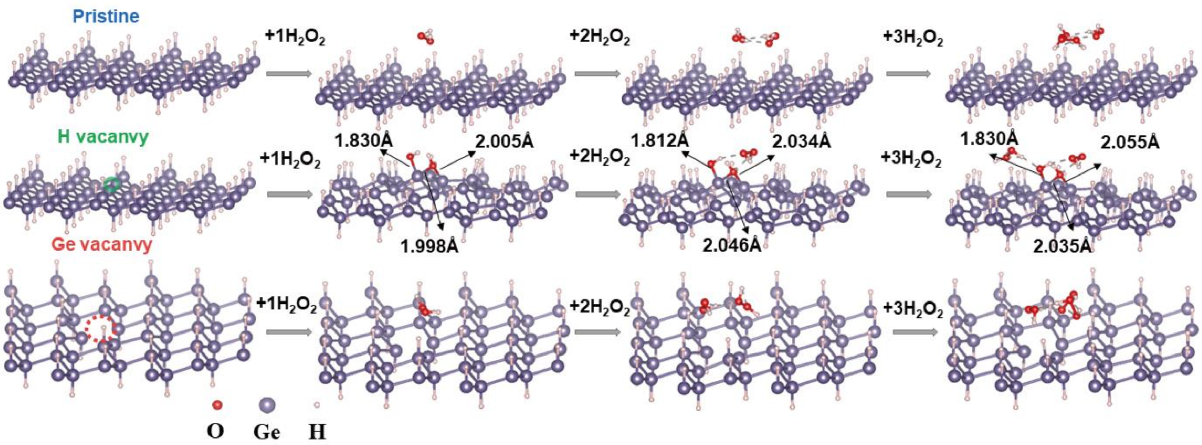

Figure S3. The surface structure change of *p*H-germanene, H*-germanene, and H-germanene* during the scavenging process of H_2O_2.

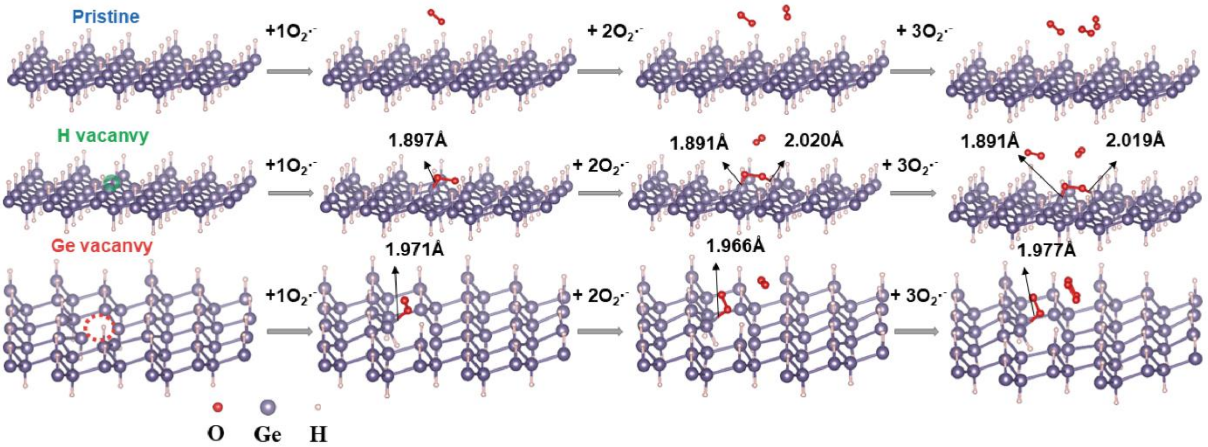

Figure S4. The surface structure change of *p*H-germanene, H*-germanene, and H-germanene* during the scavenging process of $O_2^{\bullet-}$.

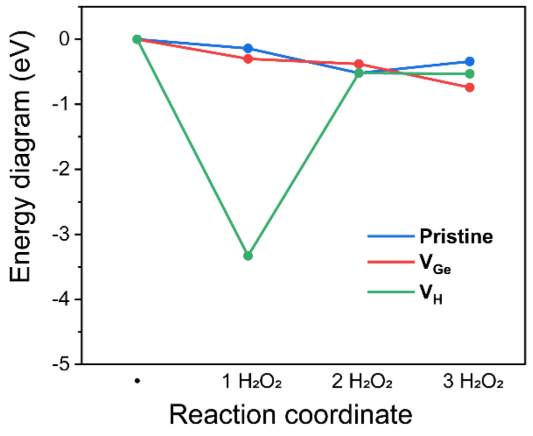

Figure S5. The energy diagram of *p*H-germanene, H*-germanene, and H-germanene* during the scavenging process of H_2O_2.

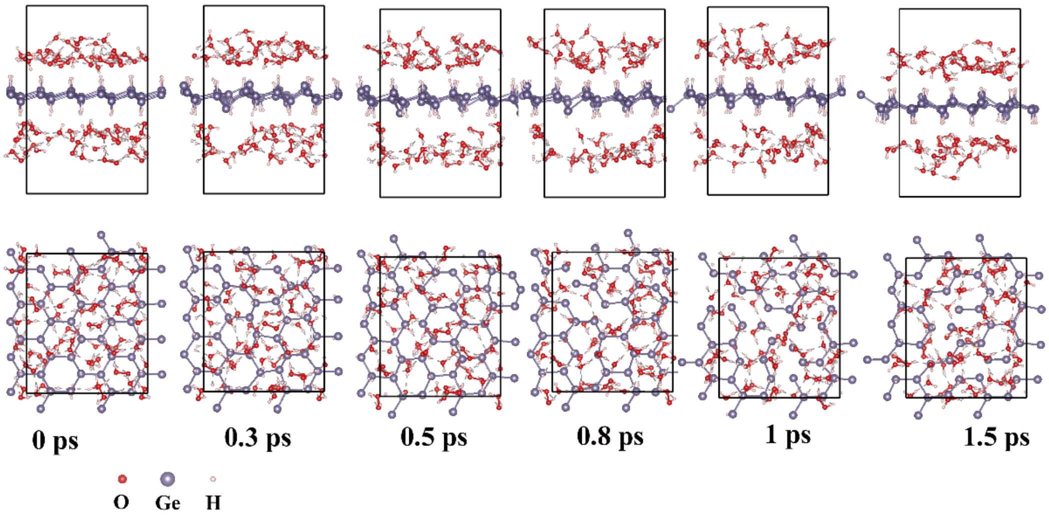

Figure S6. Molecular dynamics (MD) simulation in aqueous H_2O_2 solution with few H vacancies on the surface of H-germanene.

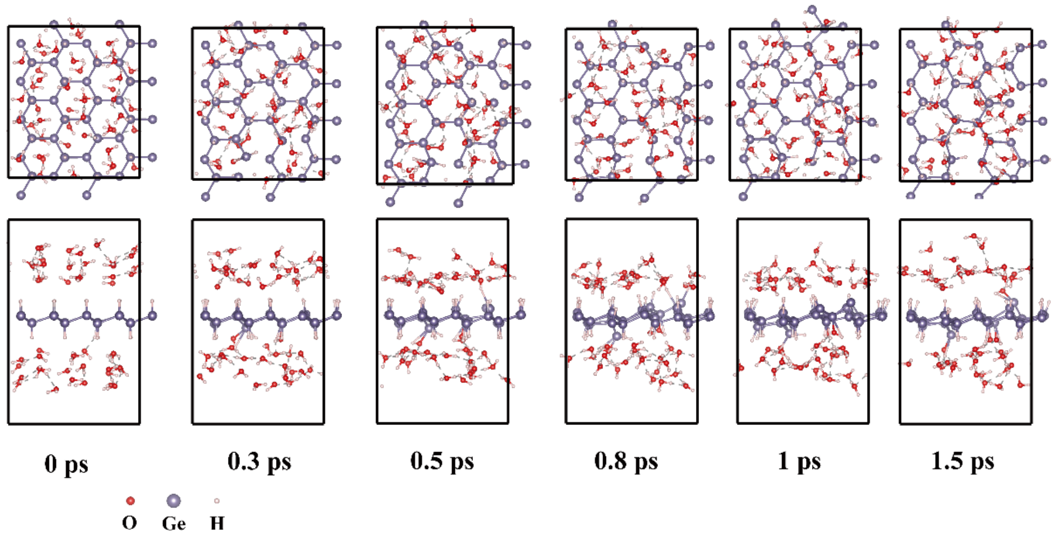

Figure S7. Molecular dynamics (MD) simulation in the solution containing ·OH when there are few H vacancies on the surface of H-germanene.

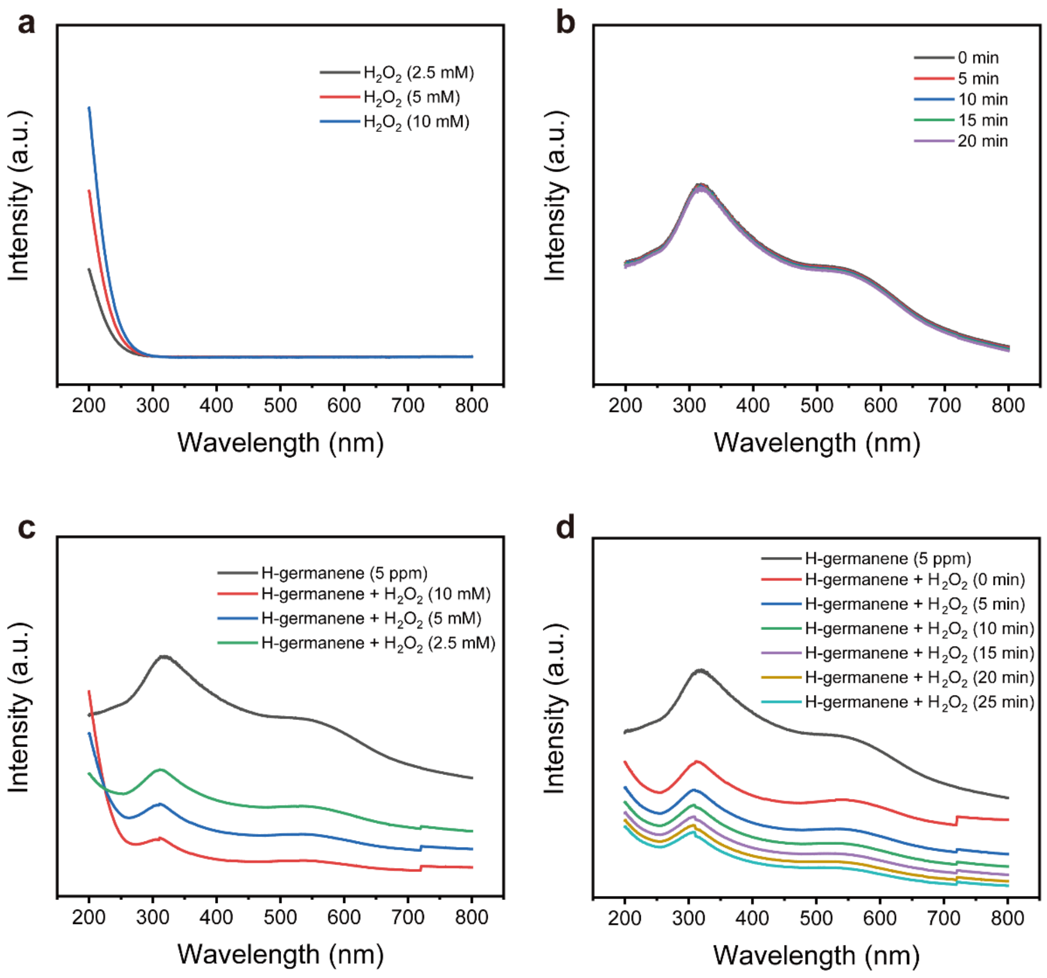

Figure S8. Absorbance spectra of different systems: (a) H_2O_2 of different concentrations, (b) H-germanene of different concentrations, (c) the systems containing H-germanene (5 ppm) and H_2O_2 of different concentrations, and (d) the systems containing H-germanene (5 ppm) and H_2O_2 (5 mM). ppm = µg mL^{-1}.

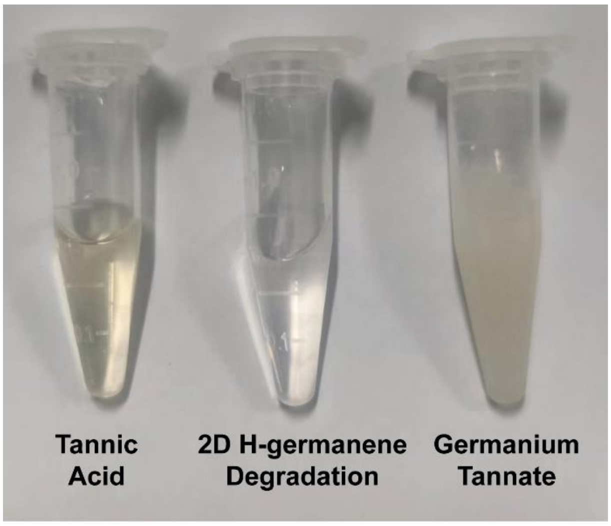

Figure S9. Identification of products after H-germanene scavenging ROS.

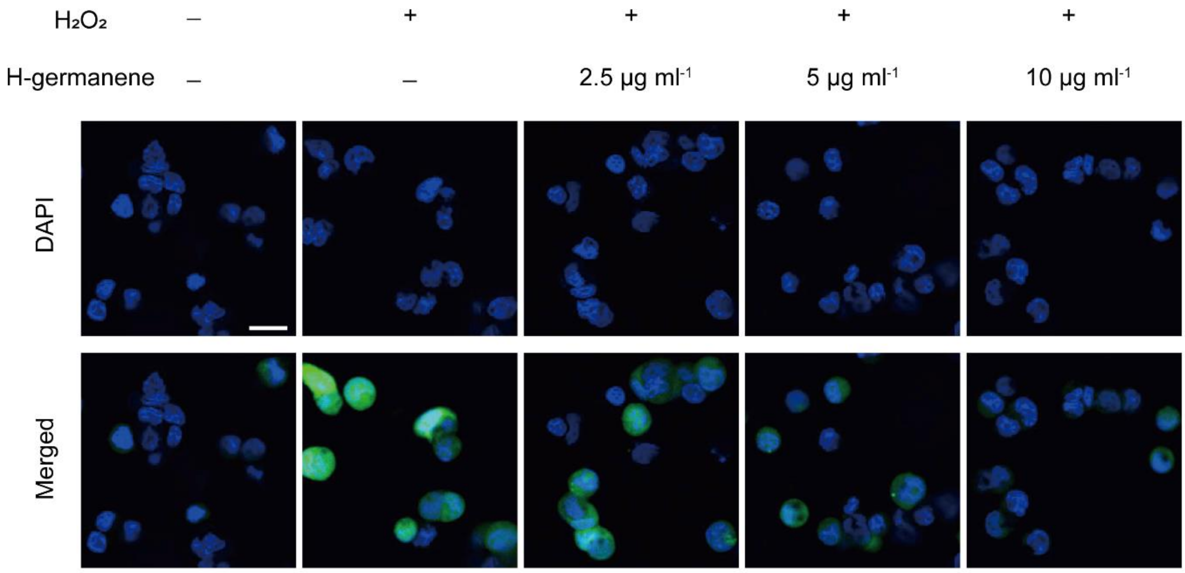

Figure S10. Confocal images of HEK293 cells under different treatments. ROS: green fluorescence; nucleus: blue fluorescence. Scale bar, 20 μm.

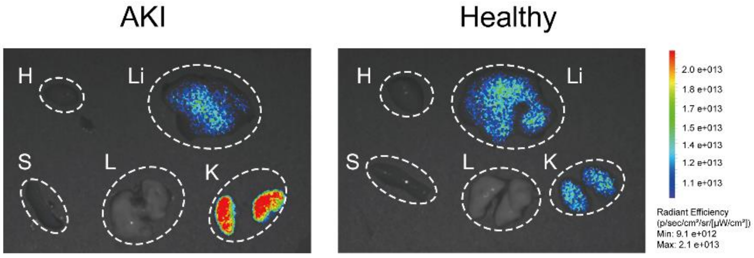

Figure S11. Fluorescence images of H-germanene in major organs (heart, liver, spleen, lung, and kidney) 12 hours after intravenous injection of H-germanene in healthy mice and AKI mice.

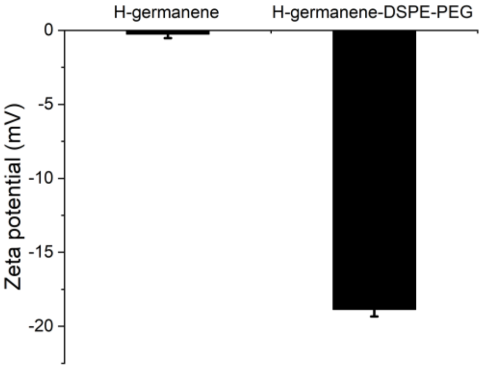

Figure S12. Zeta potential profile of H-germanene and H-germanene-DSPE-PEG dispersed in water.

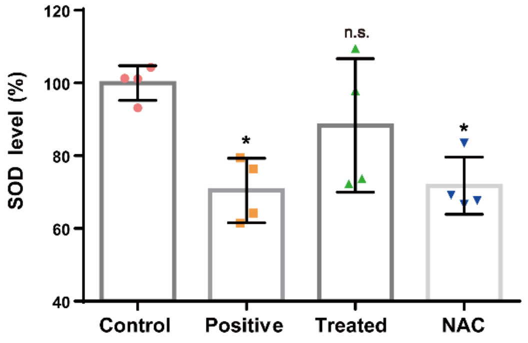

Figure S13. Renal SOD levels of mice in different groups (normal mice; AKI mice; AKI mice with intravenous injection of H-germanene; mice with intravenous injection of NAC). *P < 0.05; n.s., no significance, one-way ANOVA.

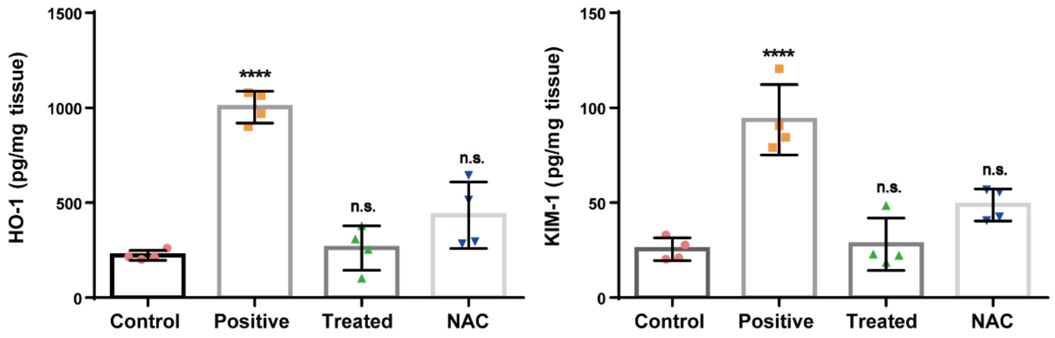

Figure S14. Renal HO-1 and KIM-1 levels of mice in different groups (normal mice; AKI mice; AKI mice with intravenous injection of H-germanene; mice with intravenous injection of NAC). ****P < 0.0001; n.s., no significance, one-way ANOVA.

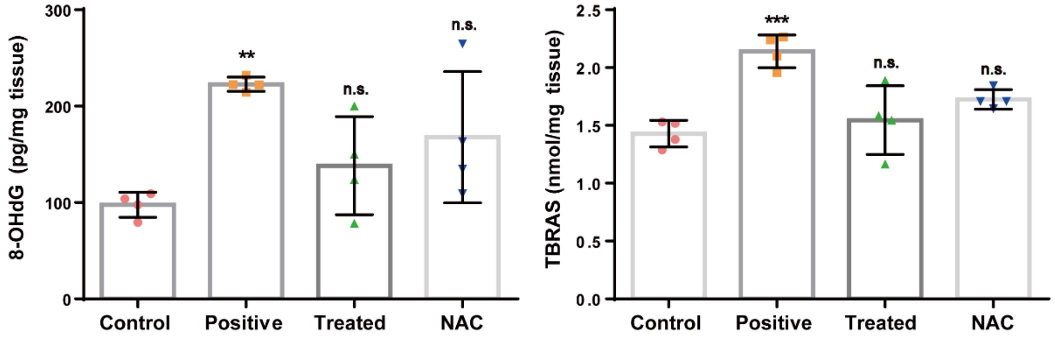

Figure S15. Renal 8-OHdG and TBRAS levels of mice in different groups (normal mice; AKI mice; AKI mice with intravenous injection of H-germanene; mice with intravenous injection of NAC). ***P < 0.001; **P < 0.01; n.s., no significance, one-way ANOVA.

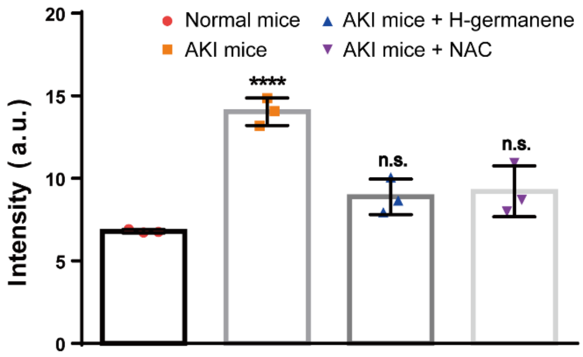

Figure S16. Quantitative statistics of superoxide in kidney slices of different groups.

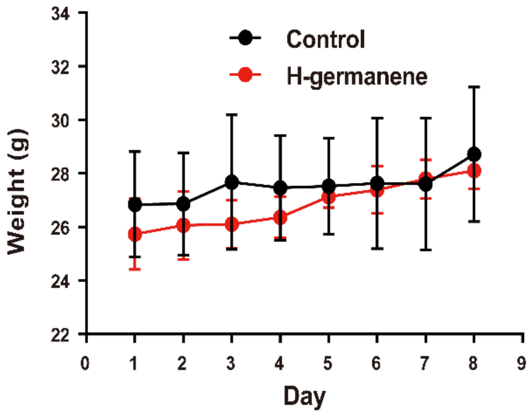

Figure S17. Body weight curve of mice after intravenous injection of H-germanene and PBS during seven days. Data represent means ± SD (N = 3).

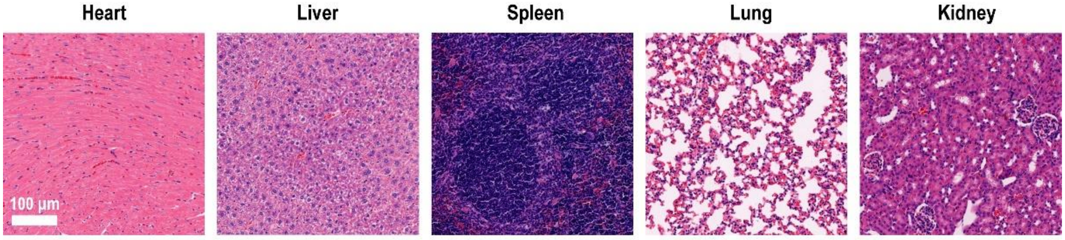

Figure S18. H&E staining of major organs (heart, liver, spleen, lung, and kidney) in healthy mice at 7 days after intravenous injection of H-germanene.
